# STRENGTHENING CAREGIVER SUPPORT: ASSESSING AND ADDRESSING CAREGIVERS’ MENTAL HEALTH NEEDS

**DOI:** 10.1093/geroni/igae098.1235

**Published:** 2024-12-31

**Authors:** Christine Gould, Marika Humber, Ranak Trivedi

**Affiliations:** Chair; Co-Chair; Discussant

## Abstract

Family caregivers (typically patients’ unpaid relatives/friends) are vital to patient care, but their mental health needs are often overlooked. This may be exacerbated by the fact that the caregiving role can be isolating, stressful, and time-consuming. This symposium highlights promising findings related to the identification of caregiver mental health and well-being needs and potential interventions in the community and Veterans’ Health Administration (VHA). First, Dr. Moo will present findings from a national survey of caregivers of older Veterans that shows the impact of care responsibilities, health care navigation, and environment on caregiver quality of life. Second, Dr. Van Orden will describe her investigation of suicide ideation, perceived burden, and social connection among caregivers of persons with dementia. Third, Dr. Humber will describe the impact of a tele-geriatric psychiatry service in two VHA regions on caregivers of rural-dwelling older Veterans with neurocognitive and or psychiatric disorders. Last, Dr. VanPutten will discuss adaptations of Dialectical Behavior Therapy (DBT) for caregivers in high-conflict caregiving situations with persons with neurocognitive disorders. She will describe adaptations made to a DBT skills training group related to the influence of caregiver physical health problems on detecting and applying core DBT skills. Dr. Ranak Trivedi, the national Training Director of the Elizabeth Dole National Center of Excellence for Veteran and Caregiver Research and member of the Research Collaborative of the National Alliance of Caregiving, will close with discussion of the presentations and will highlight opportunities for future caregiver mental health research.

